# 伴t(7;11)(p15;p15)/NUP98-HOXA9急性髓系白血病3例报告并文献复习

**DOI:** 10.3760/cma.j.issn.0253-2727.2022.02.011

**Published:** 2022-02

**Authors:** 朕豪 张, 伟 万, 玲娣 李, 菲 董, 其辉 李, 艳芳 王, 文丽 万, 化 王, 晶 王, 红梅 景

**Affiliations:** 1 北京大学第三医院血液科，北京 100191 Department of Hematology, Peking University Third Hospital, Beijing 100191, China; 2 北京大学人民医院、北京大学血液病研究所、国家血液系统疾病临床医学研究中心、造血干细胞移植北京市重点实验室，北京 100044 Peking University People's Hospital, Peking University Institute of Hematology, National Clinical Research Center for Hematologic Disease, Beijing Key Laboratory of Hematopoietic Stem Cell Transplantation, Beijing 100044, China

核孔蛋白98（Nucleoporin 98，NUP98）基因位于11号染色体短臂末端的1区5带上，当发生累及11p15的平衡易位或倒位时，导致NUP98基因重排，截至目前已发现至少28种与之相关的伙伴基因[1]，以涉及同源异型盒（Homeobox，Hox）基因家族最为多见，涵盖了HOXA（7p15）、HOXB（17q21）、HOXC（12q13）、HOXD（2q31）四个基因簇，其中，由t（7;11）（p15;p15）形成NUP98-HOXA9融合基因是NUP98基因重排最常见的形式，以在急性髓系白血病（AML）中发生为主，发生率约为2.2％[2]–[3]，该融合基因参与诱导白血病的发生，且与不良预后密切相关。我们回顾性分析了我院2017–2020年发现的3例伴有t（7;11）（p15;p15）且NUP98-HOXA9融合基因阳性AML-M_2_患者的临床和实验室资料，并进行相关文献复习。

## 病例资料

例1，男，45岁，主诉头晕、头痛伴乏力3周。入院血常规：WBC 159.42×10^9^/L，RBC 1.07×10^12^/L，HGB 41g/L，PLT 23×10^9^/L；血浆生化：LDH 1567 U/L；骨髓细胞形态学：有核细胞增生明显活跃，粒红比为61.33。粒系占92.0％，原始粒细胞占40.0％，早幼粒细胞占25.0％，偶见Auer小体。红系占1.5％，成熟红大小不等。淋系、单核系均见无殊。全片仅见2个颗粒型巨核细胞，片中散在血小板少见。过氧化物酶（POX）染色：原始幼稚细胞阳性；流式细胞术免疫表型：34.41％为异常早期髓系细胞，表达CD34、CD117、CD64、CD33、CD13、HLA-DR、CD123、CD38、cMPO, 部分表达CD11b、CD11c、CD56, 不表达CD7、CD14、CD15、CD16、CD4、CD3、CD19、CD79a、cCD3。染色体核型: 46, XY, t（7;11）（p15;p15）[19]/46,XY[1]。FISH：NUP98基因双色分离探针重排阳性百分率为85％。NUP98-HOXA9融合基因定性阳性，WT1定量：24.45％。二代测序（NGS）：RUNX1基因p.T354fs位点缺失突变（14.96％）、GATA2基因p.A372T位点突变（33.61％）、FLT3-ITD位点插入突变。诊断为AML-M_2a_。予以DA（柔红霉素、阿糖胞苷）方案1个疗程后取得完全缓解（CR），后分别行DA、MA（米托蒽醌、阿糖胞苷）方案各1个疗程，期间均处于CR状态，7个月后首次复发，尝试CAG（阿糖胞苷、阿克拉霉素、G-CSF）、MA+地西他滨、小剂量AA（阿克拉霉素、阿糖胞苷）、HA（高三尖杉酯碱、阿糖胞苷）、HAD（HA+柔红霉素）等方案均未缓解，患者于首次复发14个月后死亡。

例2，男，72岁，主诉发热伴心悸、头晕、头痛2周。入院血常规：WBC 105.18×10^9^/L，RBC 1.34×10^12^/L，HGB 48 g/L，PLT 18×10^9^/L；血浆生化：LDH 640 U/L；骨髓细胞形态学：有核细胞增生极度活跃，粒红比为11.4。粒系占85.5％，原始粒细胞占62.5％，早幼粒细胞占10.0％，偶见Auer小体。红系占7.5％，各期可见，成熟红大小不等。淋系、单核系、浆细胞均见无殊。巨核细胞及血小板少见。POX染色：原始幼稚细胞阳性；流式细胞术免疫表型：75.89％为异常早期髓系细胞，表达CD34、CD117、CD33、CD123、CD13、CD38、cMPO、CD58, 部分表达HLA-DR、CD11c、CD64, 不表达CD16、CD15、CD11b、CD10、CD14、CD3、CD4、CD5、CD8、cCD3、CD19、CD20、CD79a、CD22、CD7、CD56。染色体核型: 47,XY,t（7;11）（p15;p15）,+8[18]/46,XY[2]。FISH: NUP98基因双色分离探针重排阳性百分率为80％。NUP98-HOXA9融合基因定性阳性，WT1定量51.83％。未进行NGS检测。诊断为AML-M_2a_。予以小剂量AA方案（阿克拉霉素 10 mg第1～13天，阿糖胞苷 50 mg第1～13天）化疗1个疗程，骨髓穿刺（骨穿）评估为部分缓解（PR）后出院返当地医院治疗，电话随访确认患者于离院1个月后死亡。

例3，女，45岁，主诉乏力、头晕、心悸8个月余，确诊AML1周。3个月前以“全血细胞减少待查”就诊于外院。骨髓细胞形态学：增生减低，原始细胞10.5％，可见Auer小体；外周血原始细胞12％；骨髓活检：增生较活跃，约60％，粒红比例大致正常，粒系幼稚细胞增多，红系细胞核左移，巨核细胞增多，异型明显，MF-2级；流式细胞术检测示髓系原始细胞比例增高，粒系比例减低，红系部分细胞CD36、CD71表达减弱或缺失；染色体核型：46,XX,t（7;11）（p15;p15）[6]；未做融合基因检测。综上诊断为骨髓增生异常综合征伴原始细胞增多（MDS-EB）-Ⅱ，未进一步治疗。2个月前就诊于另一外院，仍考虑为MDS-EB-Ⅱ，建议骨髓移植。近一个月规律复查血常规，一周前再次就诊于该院，骨髓象提示转化为AML，未做染色体核型分析，但分子生物学检出NUP98-HOXA9融合基因。为进一步治疗就诊于我院，入院血常规：WBC 3.83×10^9^/L，RBC 2.2×10^12^/L，HGB 68 g/L，PLT 22×10^9^/L；血浆生化：LDH 256 U/L；骨髓细胞形态学：有核细胞增生减低，粒红比为3.87。粒系占60.0％，原始粒细胞占30.0％，早幼粒细胞占2.0％，偶见Auer小体。红系占15.5％，各期皆见，成熟红细胞大小不等。单核细胞占5.0％，幼稚单核细胞占2.0％。淋系占19.5％，无殊。巨核细胞未见，片中散在血小板少见。POX染色：原始幼稚细胞阳性率大于3％；流式细胞术免疫表型：26.91％为异常早期髓系细胞，表达CD117、CD34、HLA-DR、CD123、CD33、CD13、CD38、cMPO^dim^，部分表达CD11c, 少量表达CD71, 不表达CD10、CD15、CD14、CD64、CD16、CD11b、CD56、CD61、CD36、CD7、CD3、CD19、CD5、CD20、cCD3、cCD79a。染色体核型：46,XX,t（7;11）（p15;p15）[20]。FISH：NUP98基因双色分离探针重排阳性百分率为86％（图1）。染色体微阵列分析（CMA）：11号染色体11p13区带处存在562 kb缺失，异常区带及基因组坐标（ISCN 2016）为arr［GRCh37］11p13（32301827-32863471）×1，该处含有5个OMIM基因：WT1（607102）、WT1-AS（607899）、EIF3M（609641）、CCDC73（612328）、PRRG4（611690）。NUP98-HOXA9融合基因定性阳性，WT1定量 12.363％。NGS检出NRAS基因p.G13R rs121434595非同义突变（11.23％）、WT1基因p.V379fs移码型插入突变（21.49％）。诊断为AML-M_2a_。予以地西他滨+CAG方案，化疗第10天因出现粒细胞缺乏、发热（体温39 °C）停止化疗，后出现意识障碍，转至RICU经气管插管使用呼吸机抢救，后家属放弃治疗，于离院5 d后死亡。

**图1:**
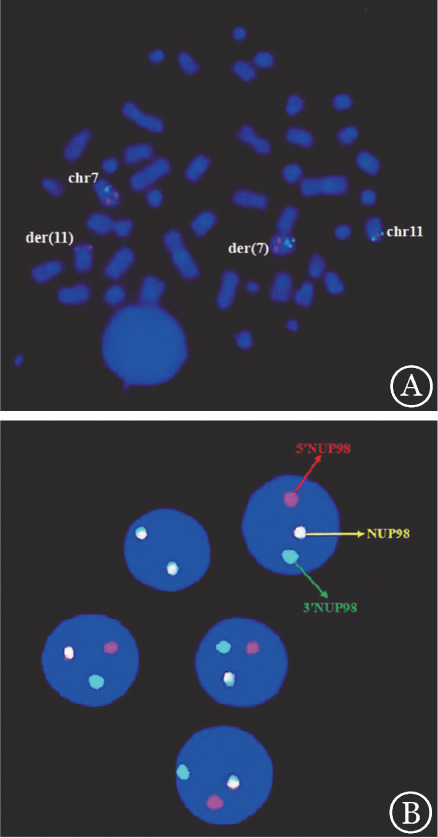
例3患者中期及间期FISH结果 A：7号染色体着丝粒（CEP7，绿色）/D7S486基因（7q31，红色）位点特异探针用于明确定位正常及衍生7号染色体同时区分7号染色体长、短臂；NUP98（11p15.4）基因近着丝粒端（5′NUP98，132 kb）标记为红色，近端粒端（3′NUP98，151 kb）标记为绿色，中期分裂象内可见NUP98基因重排阳性；B：间期细胞核内NUP98基因呈现典型重排阳性信号特征（正常信号特征为2F，典型重排阳性信号特征为1R1G1F；R为红色信号，G为绿色信号，F为红绿叠加的黄色信号）

## 讨论及文献复习

自1982年Tomiyasu等[4]首次发现以来，截至目前，伴有 t（7;11）（p15;p15）且NUP98-HOXA9融合基因阳性的血液肿瘤在国内外已累计报道了至少229例（不包含本研究的3例），可见于MDS、慢性髓性白血病（CML）加速期/急变期、急性白血病（包括novo AML、t-AML、T-ALL、MAL）、治疗相关髓系肿瘤，中位生存期仅8～13个月[3]，由于发病率较低，多以个案形式报道，在有限的大宗病例中，时间跨度较大，且多数未进行深入的细胞及分子遗传学研究，伴有此异常的AML以M_2_多见，M_4_偶见，在流行病学方面，其发病具有明显的地域及种族差异，主要体现为东亚人群的发病率显著高于西方人群[5]–[7]，在东亚以日本报道病例最多，性别分布以女性居多，常表现为多系病态造血，LDH明显增高，形态学可见Auer小体[8]，NAP积分减低[9]，流式细胞术免疫表型以CD13、CD33、CD34、CD38、CD117、CD123、cMPO、HLA-DR同时阳性表达多见，本研究纳入的3例患者与这些既往报道的临床特征基本相符，在性别分布方面的偏差则可能与本研究病例数有限有关。

在细胞遗传学特征方面，伴NUP98-HOXA9融合阳性患者的核型除了可出现单一t（7;11）（p15;p15）的克隆性异常外，还可伴随出现数目异常如+8、+Y，或伴随结构异常如t（9;22）（q34;q11）、t（9;12）（q34;p13）、t（1;21）（p32;q22）、t（5;12）（q33;p13）、add（6）（p25）、del（12）（p12），此外一些变异易位如t（7;11;13;17）同样可导致NUP98-HOXA9融合基因形成，关于合并上述异常或t（7;11）变异易位（NUP98-HOXA9阳性）与单纯t（7;11）（p15;p15）/NUP98-HOXA9阳性患者的治疗与预后比较仍需积累更多病例才可知。

在分子生物学特征方面，基础研究和临床研究均已发现伴NUP98-HOXA9的白血病相较其他白血病有其独特之处。Mayotte等[10]发现NUP98-HOXA9与BCR-ABL1共表达时CML-CP小鼠模型在7～10 d内转化为急性白血病，而单一NUP98-HOXA9或BCR-ABL1表达的小鼠模型超过2个月仍未出现急变，证实了NUP98-HOXA9与BCR-ABL1在CML急变中的协同促进作用，Dash等[11]也同样佐证了上述观点。另外，WT1、KRAS、NRAS基因突变也与NUP98-HOXA9融合基因具有显著相关性[12]–[14]，这三种基因的突变可能参与协同NUP98-HOXA9融合基因导致白血病的发生。此外，NUP98-HOXA9融合基因阳性时更易伴随FLT3-ITD突变 [15]。本研究的患者中未检出KRAS基因突变，但例3存在WT1基因突变并伴有NRAS基因突变，例1检出RUNX1基因、GATA2基因、FLT3-ITD基因突变，除WT1、NRAS、FLT3-ITD基因以外检出的余下2种基因突变是否与NUP98-HOXA9融合基因相关目前未知。除了上述三种已经明确的基因突变与NUP98-HOXA9融合基因相关外，由于WT1（11p13）基因与NUP98（11p15）基因邻近，当发生t（7;11）（p15;p15）时，NUP98基因重排是否会引起邻近WT1基因的高表达、突变或重排目前尚需证实。

本研究中例3经CMA发现11p13处存在562 kb缺失，在所受累的五个基因中，WT1、WT1-AS两个基因已在血液肿瘤及多种恶性肿瘤中报道过，而其余三个基因（EIF3M、CCDC73、PRRG4）在血液肿瘤中还未见报道。结合NGS的结果，提示例3 WT1基因为突变与缺失并存的异常，即WT1双等位基因的失活，这与单缺失或单突变的结局相比更差，我们猜测这可能是造成该例患者快速进展及疗效较差的原因之一。

从治疗角度而言，目前尚无针对t（7;11）（p15;p15）/NUP98-HOXA9的靶向药物或统一标准的治疗方案，此类患者仅从单纯传统化疗中的获益程度有限，异基因造血干细胞移植依旧是目前克服或改善NUP98-HOXA9所致不良预后的首选方法，但由于受到供者来源、患者的年龄及化疗缓解程度等因素影响，使得此类患者并非均能接受移植治疗，此时，研发及寻找潜在靶向药物尤为必要。Rio-Machin等[16]发现组蛋白去乙酰化酶抑制剂（HDACi）的一种代表性药物——帕比司他对表达NUP98-HOXA9的人类造血祖细胞（hHP）模型具有强抑制作用，并且这种抑制作用明显强于抑制表达MLL-AF9或表达RUNX1-RUNX1T1的hHP，因此，帕比司他有望今后成为治疗NUP98-HOXA9阳性AML的新型靶向药物。然而该药物尚未在中国上市，目前，西达本胺作为国内患者唯一可及的HDACi类药物，已经在难治复发AML患者中体现出了良好的安全性和有效性，以其为代表的CDCAG方案已证实能使难治复发AML患者得到更高的缓解率且耐受性较好[17]，但现仍缺乏初治患者的疗效研究。另外，CDK6已被证实是NUP98融合蛋白的关键直接靶标[18]，因此CDK4/6抑制剂有望在未来成为伴有NUP98融合蛋白AML患者的一个新的治疗选择。现阶段国内一项研究表明伴NUP98基因重排的老年及不耐受标准诱导治疗的患者应用含去甲基化药物治疗可获得较好的疗效[19]。

从检测角度来看，应用qRT-PCR监测NUP98-HOXA9融合基因的MRD尤为必要，有助于临床及时了解是否发生分子学复发，尽早干预。另外，当染色体检出t（7;11）（p15;p15）时并非一定形成NUP98-HOXA9融合基因，也有可能形成更加罕见的NUP98-HOXA11或NUP98-HOXA13融合基因，而普通核型受分裂象质量及显带分辨率影响，难以精确定位NUP98基因的相关融合，目前对于NUP98-HOXA9融合基因主要依赖于qRT-PCR检出，值得注意的是，由于NUP98基因的伙伴基因众多，而qRT-PCR仅能检测现有已知的相关融合基因，因此，在实际工作中联合应用染色体核型、FISH、qRT-PCR将有助于NUP98基因重排及其产物的检出，FISH相较PCR而言不受引物设计局限的影响，在今后鉴定新型或隐匿性NUP98基因/11p15.4重排中具有独特优势。
